# A Mobile App for Self-management of Urgency and Mixed Urinary Incontinence in Women: Randomized Controlled Trial

**DOI:** 10.2196/19439

**Published:** 2021-04-05

**Authors:** Towe Wadensten, Emma Nyström, Karin Franzén, Anna Lindam, Elisabet Wasteson, Eva Samuelsson

**Affiliations:** 1 Family Medicine, Department of Public Health and Clinical Medicine Umeå University Umeå Sweden; 2 Women’s Clinic Örebro University Hospital Örebro Sweden; 3 School of Medical Sciences Örebro University Örebro Sweden; 4 Unit of Research, Education, and Development, Östersund Hospital Department of Public Health and Clinical Medicine Umeå University Umeå Sweden; 5 Department of Psychology and Social Work Mid Sweden University Östersund Sweden

**Keywords:** eHealth, mHealth, urinary incontinence, urgency urinary incontinence, mixed urinary incontinence, self-management, mobile app, smartphone app, women

## Abstract

**Background:**

Many women experience urgency (UUI) and mixed (MUI) urinary incontinence but commonly hesitate to seek care. Treatment access and self-management for these conditions can be supported through eHealth approaches.

**Objective:**

This study aimed to investigate the efficacy of the mobile app Tät II for self-management of UUI and MUI in women.

**Methods:**

This randomized controlled trial included women ≥18 years old with UUI or MUI and ≥2 leakages per week. Those with red-flag symptoms were excluded. Participants were recruited via analog and digital advertisements and screened for initial selection through a web-based questionnaire. Data were collected using another questionnaire and a 2-day bladder diary. A telephone interview confirmed the symptom diagnosis. Participants were randomized (1:1) to receive access to a treatment app (including pelvic floor muscle training, bladder training, psychoeducation, lifestyle advice, tailored advice, exercise log, reinforcement messages, and reminders) or an information app (control group), with no external treatment guidance provided. The primary outcome was incontinence symptoms at the 15-week follow-up, measured using the International Consultation on Incontinence Questionnaire (ICIQ)−Urinary Incontinence Short Form (ICIQ-UI SF). Urgency symptoms were assessed using the ICIQ−Overactive Bladder Module (ICIQ-OAB) and quality of life using the ICIQ−Lower Urinary Tract Symptoms Quality of Life Module (ICIQ-LUTSqol). Incontinence episode frequency (IEF) was calculated per bladder diary entries. Improvement was measured using the Patient’s Global Impression of Improvement. All outcomes were self-reported. Cure was defined as no leakages per the bladder diary. Intention-to-treat analysis was performed.

**Results:**

Between April 2017 and March 2018, 123 women (mean age 58.3, SD 9.6 years) were randomized to the treatment (n=60, 2 lost to follow-up) or information (n=63) group. Of these, 35 (28%) women had UUI, and 88 (72%) had MUI. Mean ICIQ-UI SF score at follow-up was lower in the treatment group than in the information group (estimated difference −3.1, 95% CI −4.8 to −1.3). The estimated between-group difference was −1.8 (95% CI −2.8 to −0.99) for mean ICIQ-OAB score and −6.3 (95% CI −10.5 to −2.1) for the mean ICIQ-LUTSqol score at follow-up. IEF reduction from baseline to follow-up was greater in the treatment group (−10.5, IQR −17.5 to −3.5) than in the information group (*P*<.001). Improvement was reported by 87% (52/60) of treatment group participants and by 30% (19/63) of information group participants. The cure rate was 32% in the treatment group, and 6% in the information group (odds ratio 5.4, 95% CI 1.9-15.6; *P*=.002). About 67% (40/60) of the treatment group participants used the app more than thrice a week.

**Conclusions:**

The treatment app was effective for improving urgency and mixed incontinence in women. When self-management is appropriate, this app may be a good alternative to pharmacological treatment or other conservative management, thus increasing access to care.

**Trial Registration:**

ClinicalTrials.gov NCT03097549; https://clinicaltrials.gov/ct2/show/NCT03097549

## Introduction

Urinary incontinence is a common problem that affects many women at some time during their lives, with reported prevalence rates ranging between 25% and 45%, depending on the population and study design [1-4]. There are several types of urinary incontinence. Stress urinary incontinence (SUI) is defined as leakage upon exertion (eg, during coughing or jumping), urgency urinary incontinence (UUI) involves urinary leakage combined with an urge to void, and mixed urinary incontinence (MUI) manifests as a combination of SUI and UUI symptoms [5]. Prevalence rates vary from 1% to 11% for UUI and from 2% to 36% for MUI [1,4]. Overactive bladder is a broad term that includes UUI, and it is defined as the experience of a compelling urgency to void, often combined with more frequent voiding, and sometimes nocturia [5]. These conditions can lead to a sense of shame, social isolation, and lower self-esteem—with a significant impact on health-related quality of life [6-8].

For the three main types of urinary incontinence, the recommended first-line treatment includes pelvic floor muscle training (PFMT) and, where appropriate, lifestyle changes (eg, reduced caffeine intake, modified fluid intake, and weight reduction if overweight) [9,10]. Unsupervised PFMT has been recommended in cases wherein an underlying pathology is absent [9]. For women with urgency-predominant urinary incontinence and small micturition volumes, bladder training is recommended, with scheduled voiding or prolonged voiding intervals [3,9,10]. According to a recent review, PFMT might also be useful in overactive bladder treatment, but more evidence is needed in this regard [11]. As a second line of treatment, pharmacological therapy is recommended and widely used, but it often exhibits only modest effectiveness and commonly leads to side effects [3,10].

Although effective treatments for urinary incontinence are available, they do not cater to all individuals who may benefit from them [9,12]. Some studies describe patients’ reluctance to seek help for urinary incontinence, sometimes explained by a sense of embarrassment or mistrust in health care [6,13]. Self-management or treatment options that do not require face-to-face contact might be suitable ways to provide care in some of these cases. Web-based platforms and smartphone apps represent an increasingly common way of supporting self-management or providing treatment for various conditions [14-16]. In the context of the current COVID-19 pandemic, the interest in these kinds of technical solutions has increased even further, and urology is one such field where technology-supported treatment or self-management might be useful [17,18]. However, among the currently available treatment apps related to urinary incontinence, only few have been evaluated with regard to their efficacy [19].

As part of the current research project, we examined the effects of an internet-based treatment program and a smartphone app designed for women with SUI. Both programs were found to be effective treatment options with regard to short-term and long-term improvement of clinically relevant symptoms as well as cost-effectiveness [20-24]. However, evidence regarding app-based treatment for women with UUI or MUI remains scarce. Since urgency-predominant urinary incontinence may be associated with an underlying disease, physical examination is recommended before treatment. An algorithm comprising structured questions combined with dipstick urinalysis has been found to be useful in identifying women who may benefit from management in ways other than the usual care provided [25]. Along with other innovative options for providing non−face-to-face diagnosis and treatment for UUI and MUI, this approach might facilitate patients to seek help regarding urinary incontinence and enable increased access to treatment.

We have developed a new smartphone app featuring a complex, individually tailored treatment program designed to help patients self-manage UUI and MUI. In this study, we aimed to evaluate whether this app was effective for improvement and cure of UUI and MUI in women.

## Methods

### Study Design and Participants

This 1:1 randomized, controlled, parallel-arm trial was performed in Sweden between April 2017 and September 2018. Community-dwelling adult women were recruited via information broadcasted on TV, radio, and newspapers in Sweden, and via targeted Facebook advertisements. The inclusion criteria were as follows: female sex, age ≥18 years, experiencing UUI or MUI with ≥2 leakages/week and symptoms lasting for ≥12 months, access to a smartphone (at least iOS 8.0 or Android 4.0.3), and the ability to send and receive email. The exclusion criteria were as follows: pregnancy, use of another PFMT app, use of mirabegron or antimuscarinic drugs, and incontinence surgery within the last 5 years. Additional exclusion criteria related to red-flag symptoms and certain medical conditions were also considered, namely, painful urgency; previous pyelonephritis; ≥3 urinary tract infections in the last 12 months; dysuria (burning sensation when voiding); visible hematuria; noninvestigated bladder-emptying difficulties; metrorrhagia; cancer in the pelvic area, bladder, or bowels; decreased mobility or sensitivity in the legs or pelvic area; history of stroke; neurological disease; or diabetes.

Initial selection was performed using a web-based screening questionnaire that included questions regarding education level, postal code, and inclusion and exclusion criteria, which was available on the eContinence project website. To distinguish sex from gender, the question “Are you a woman?” was followed by the question “Were you assigned female sex at birth?” To identify red flags, an algorithm containing structured questions about the presence of relevant symptoms was integrated into the questionnaire (Multimedia Appendix 1). This algorithm was developed through several workshops with researchers and clinicians, and it was based on the best available evidence and clinical experience. Respondents presenting any red flags or other exclusion criteria were not allowed to proceed with the questionnaire, and they were automatically recommended to seek usual care (ie, contact their ordinary health care provider). After completing the full questionnaire and submitting their email address, eligible respondents received an email with an informed consent form and a printable 2-day bladder diary. Respondents with a maximum voided volume of ≤150 mL were deemed ineligible and were contacted by a physician (ES) who redirected them to their usual health care provider as a precautionary measure. The remaining respondents completed a web-based inclusion questionnaire comprising items regarding background information, medical history and lifestyle, more detailed symptom questions, as well as forms related to the outcome measures. In all web-based questionnaires, respondents were required to answer each question in order to proceed to the next. Nonrespondents who did not submit their informed consent and bladder diary, or those who did not answer the inclusion questionnaire, were sent two reminders via email.

Finally, each respondent was contacted via telephone by a specialist incontinence nurse or general practitioner (ES). A telephone interview was conducted with the objectives of confirming the symptom diagnosis (ie, UUI or MUI), reconfirming the absence of exclusion criteria, and ensuring that the participant was fully informed about the study.

This study was approved by the regional ethical review board of Umeå, Sweden (registry number 2016/523-31) and registered at Clinicaltrials.gov (NCT03097549). Before and during the study, and after completion, on-site monitoring was conducted by an independent monitor. The monitor ensured study performance according to the protocol, and the collection, documentation, and reporting of data following good clinical practice and applicable ethical and regulatory requirements.

### Randomization and Blinding

The participants included in the study were randomized to one of two study groups: the treatment group or the information group. An independent administrator generated the allocation sequence and prepared 130 numbered, opaque, sealed envelopes, with assignments equally distributed between the two study groups. The study coordinator opened one envelope for each participant, in the order in which they received an email from the interviewer indicating that they were ready for randomization. The participants were not blinded to their allocation. Each participant received an email informing them of their assigned group and providing instructions on how to access the relevant app. Participants randomized to the information group were notified that they would gain access to the intervention once of their follow-up data for the trial was complete.

### Intervention and Procedures

**Figure 1:**
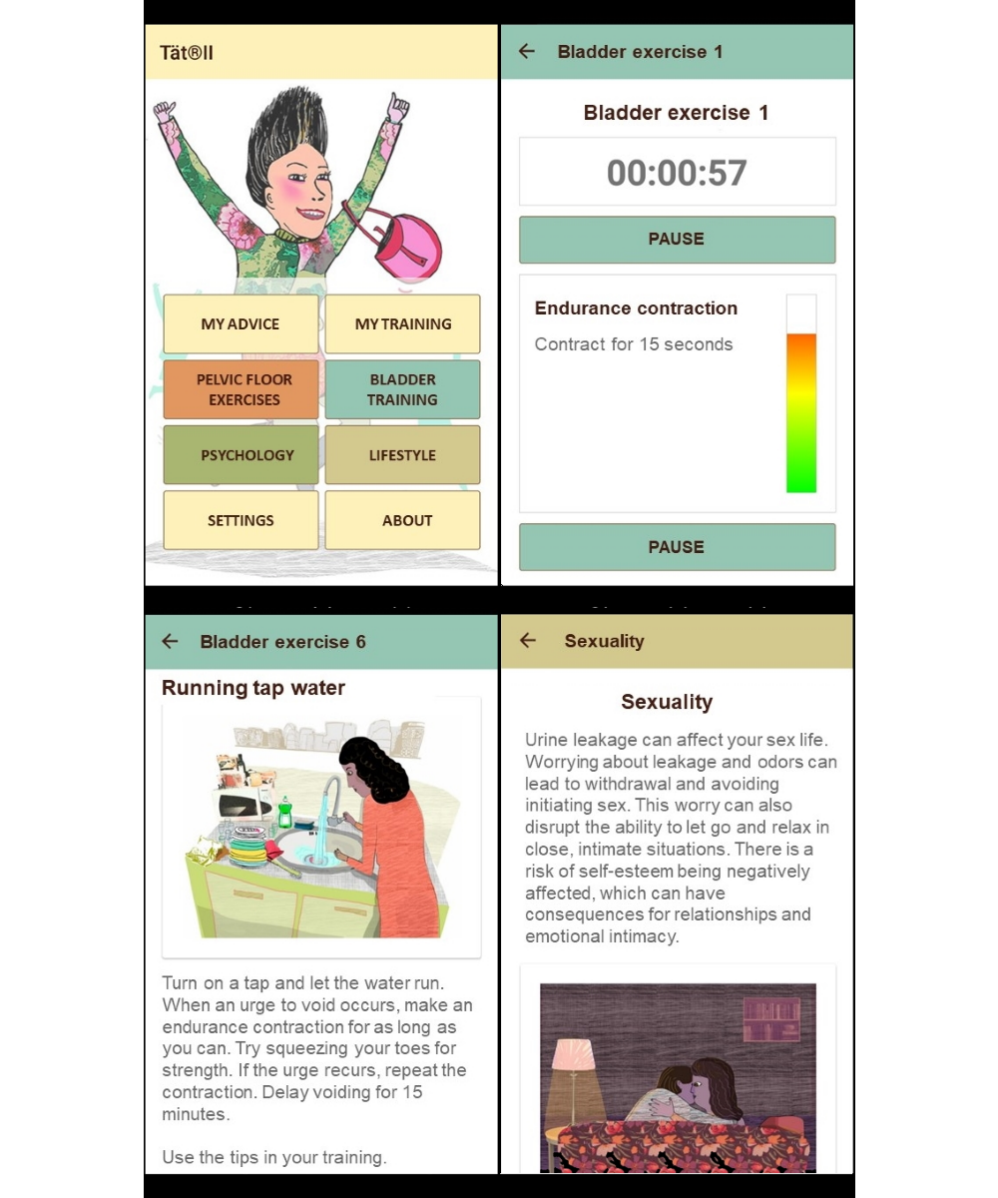
Screenshots from the treatment app (Tät II). Upper-left corner: main (home) screen; upper-right corner: active view of an exercise in the bladder training program; lower-left corner: textual description of another bladder training exercise; lower-right corner: information from the lifestyle section. Text has been translated from Swedish to English for illustration purposes.

The Tät II mobile app was designed for both Android and iOS devices. The contents of the app were developed based on research and clinical experience and were discussed in 2015-2016 with a multi-professional group comprising researchers and clinicians with expertise in family medicine, urogynecology, urology, specialized incontinence care, and psychology. The app was developed during 2016-2018 by ES and TW, in collaboration with other researchers involved in the project and the technical development division at Umeå University. The development process also incorporated user feedback—both from users of the previous app developed within this research project and from a test group of women outside the medical professions. The Tät II app is focused on four themes: PFMT, bladder training, psychoeducation, and lifestyle advice. It also contains automatic reinforcement messages and an exercise log. Tailored advice, based on information from the user’s bladder diary and responses to the inclusion questionnaire, was designed to guide the user to the features of the app that would be most relevant to her symptoms and lifestyle (eg, bladder training was recommended if the user had small micturition portions, or weight reduction was recommended if the user was overweight). The different components of the Tät II app are detailed below (Figure 1, Table 1, and Multimedia Appendices 2 and 3). The PFMT treatment program in the app has been previously described and evaluated as part of a smartphone app developed earlier [20].

**Table 1:** Contents of the Tät II treatment app and information app.

Topic	Treatment app	Information app
Pelvic floor muscle training	Extensive information on anatomy and pelvic floor muscle training A pelvic floor muscle training program in 11 steps	Very brief information on pelvic floor muscle training
Bladder training	Extensive information on bladder physiology and bladder training A bladder training program in 7 steps^a^	Very brief information on bladder training
Psychological education	Extensive information on psychological topics related to urgency symptoms Three tasks based on cognitive behavioral therapy theory	Very brief information on psychological topics related to urgency symptoms
Lifestyle advice	Information on topics of estrogen, fluid intake, physical activity, overweight, smoking, sexuality, constipation, foods, and incontinence aids	Summarized information on lifestyle advice
Reinforcement	Recurring questions on degree of bother^b^ Automatic reinforcement messages based on progress Three customizable notifications for daily reminders	N/A^c^
Other functions	Exercise log Tailored advice on what areas to focus on in the app, based on information from bladder diary and questionnaire^d^ Optional 4-digit password protection	N/A

^a^The bladder training program featured exercises for enduring urgency to achieve longer voiding intervals. It did not feature scheduled voiding.

^b^In-app questions about the degree of bother from leakage, urgency, and worry about leaking. The user is asked these questions directly after installing and activating the treatment app, and after 4, 8, and 12 weeks of intervention.

^c^Not applicable.

^d^User is provided 4-10 advices, as relevant, on the following topics: pelvic floor muscle training, bladder training, fluid intake, psychoeducation, local estrogen treatment, obesity, smoking, and constipation.

Activation of the treatment app enabled complete access to all components of the Tät II app. For activation, participants randomized to the treatment group were registered in a database stored on a secure server. A unique one-time activation code was generated and used as a password along with the user ID. The tailored advice was automatically downloaded from the database into the app during the activation process. If the app was not activated within 2 weeks, an email reminder was sent to the participant, and if it was not activated within another week, the participant was contacted via telephone and offered technical guidance. Apart from this, the participants received no guidance from the researchers during the study.

The information app is a limited version of Tät II, which is freely downloadable from app stores. It includes a short summary of lifestyle advice and brief information about the various app components (Table 1).

At the 15-week follow-up, participants were asked to complete a web-based questionnaire and a new 2-day bladder diary. The follow-up questionnaire also included the possibility to add qualitative user feedback. After collection of the follow-up data for the trial, participants in the information group received access to the full treatment app, and participants in the treatment group received information on maintenance training. The different data collection time points of the trial are detailed in Multimedia Appendix 4.

No data were transmitted from the app apart from the voluntary submission of user statistics at follow-up. Participants were encouraged to report any potential side-effects to the research team via email or telephone. Participants were also instructed to seek usual care if any red-flag symptoms appeared.

### Outcomes

The primary outcome was the between-group difference in incontinence symptom severity at follow-up, as measured using the Swedish version of a validated questionnaire: the International Consultation on Incontinence Questionnaire−Urinary Incontinence Short Form (ICIQ-UI SF) [26]. The ICIQ-UI SF includes 3 questions about the frequency and amount of urinary leakage and its effect on everyday life. The responses are summed to obtain a total score ranging from 0 to 21 points. The severity of incontinence symptoms was categorized as slight (1-5 points), moderate (6-12 points), severe (13-18 points), or very severe (19-21 points) [27].

Secondary outcomes included urgency symptoms, quality of life, and catastrophizing. The International Consultation on Incontinence Questionnaire−Overactive Bladder Module (ICIQ-OAB) includes 4 items on the frequency of day and night micturition, urgency, and urgency leakage, and the responses are summed to obtain a total score ranging from 0 to 16 points [28]. The International Consultation on Incontinence Questionnaire−Lower Urinary Tract Symptoms Quality of Life Module (ICIQ-LUTSqol) includes 19 items regarding the impact of urinary leakage on the quality of life, and the responses are summed to obtain an overall score ranging from 19 to 76 points [28]. We also used a nonvalidated score, the Incontinence Catastrophizing (IC) Scale, which was adapted from a short version of the validated Pain Catastrophizing Scale [29]. This scale was translated to Swedish by the research group by using a structured procedure. The IC Scale comprises 7 items regarding fear of leakage and urgency, and the responses are summed to obtain a total score ranging from 0 to 21 points. For all the above-mentioned scores, a reduction in the score indicates an improvement of the symptoms.

Other secondary outcomes included the number of leakages, use of incontinence aids, impression of improvement, and patient satisfaction. Incontinence episode frequency (IEF) was calculated as the number of leakages reported in a 2-day bladder diary multiplied by 3.5 to generate the weekly number of incontinence episodes. Participants were asked about their use of incontinence aids over the last 4 weeks, and they were provided 6 response options ranging from “No, never” to “Yes, more than 1 pad per day.” The Patient Global Impression of Improvement (PGI-I) is a validated questionnaire evaluating improvement, which was only used at follow-up. Participants rated their follow-up condition as compared with their pretreatment condition, using a 7-item scale with answer options ranging from “Very much better” to “Very much worse” [30]. Patient satisfaction was evaluated only in the treatment group at follow-up. This item asked whether the current treatment was perceived as sufficient, with 3 response options regarding satisfaction and intention to seek further care.

We used information from the bladder diary and follow-up questionnaire to assess cure and improvement. *Cure* was defined as no leakage episodes recorded in the bladder diary at follow-up, and *improvement* was defined as any improvement on the PGI-I.

### Performance and Adherence

At both the baseline and follow-up, participants were asked whether they perceived themselves as able to correctly perform pelvic floor contractions. At the follow-up, they were also asked to appraise their current ability to contract their pelvic floor muscles as compared with before they had access to the assigned treatment or information app, with responses ranging from “Much better” to “Much worse,” and to appraise the ability to resist urgency through a corresponding question, with similar response options.

Furthermore, the follow-up questionnaire included a question on how often the participants had used their assigned app during the study period. Response options ranged from “Never” to “Daily, three times a day, or more often.” There was also a question on whether the participant had used another incontinence app or participated in another incontinence treatment program during the study period.

### Technical Issues and User Feedback

Participants were informed that they could contact the researchers via email in case of technical problems with the app. The follow-up questionnaire allowed participants to provide qualitative feedback via nonmandatory open-ended questions about how they perceived the assigned app, in general, and the specific contents of the app. Participants in the treatment app group were also asked to rank the 6 treatment app components (tailored advice, exercise log, PFMT, bladder training, psychoeducation, and lifestyle advice) from most useful to least useful.

### Sample Size

The expected response was based on results from our previous smartphone study, and the findings of Albers-Heitner et al [20,31]. We anticipated ICIQ-UI SF improvements of 2.5 points in the treatment group and 0.9 points in the information group. This level of change has also been found to reflect a clinically important difference in women with stress urinary incontinence after treatment via eHealth [22]. Detecting this difference with 80% power, a two-sided test, and a significance level of *P*<.05 would require a sample size of 49 in each group. With an expected drop-out rate of 20%, we needed approximately 60 participants in each group. Thus, we aimed to recruit 120 women for this study.

### Statistical Analysis

For all outcome measures, we performed an intention-to-treat analysis. To analyze the ICIQ-UI SF, ICIQ-LUTSqol, ICIQ-OAB, and IC scale, we used analytical methods that accounted for all available data. We used the last observation carried forward method for IEF and incontinence aid usage, and we applied imputation of values corresponding to no change in the PGI-I.

Baseline data were described in terms of age, BMI, educational level, medication use, and all primary and secondary outcomes that were measured at the baseline. For the primary outcome, we examined the between-group difference in the mean ICIQ-UI SF score at the 15-week follow-up using a linear mixed-model analysis incorporating baseline data. For the secondary outcomes, between-group comparisons were made using a linear mixed-model analysis for the difference in mean values for continuous variables, and the Mann-Whitney *U* test for the distribution of categorical variables and for the difference in median for nonnormally distributed continuous variables. For within-group comparisons (ie, between baseline and follow-up), we used a paired *t* test for continuous variables and a Wilcoxon signed-rank test for nonnormally distributed continuous variables. For IEF, we calculated the difference between baseline and follow-up scores for each individual, presented as median and IQR values. Since IEF data were not normally distributed, they were analyzed using the Wilcoxon signed-rank test. We used a chi-square test with continuity correction to calculate the odds ratios (ORs) for cure and improvement.

All statistical analyses were performed using SPSS (version 25; IBM Corp).

## Results

### Study Flow and Participant Characteristics

**Figure 2:**
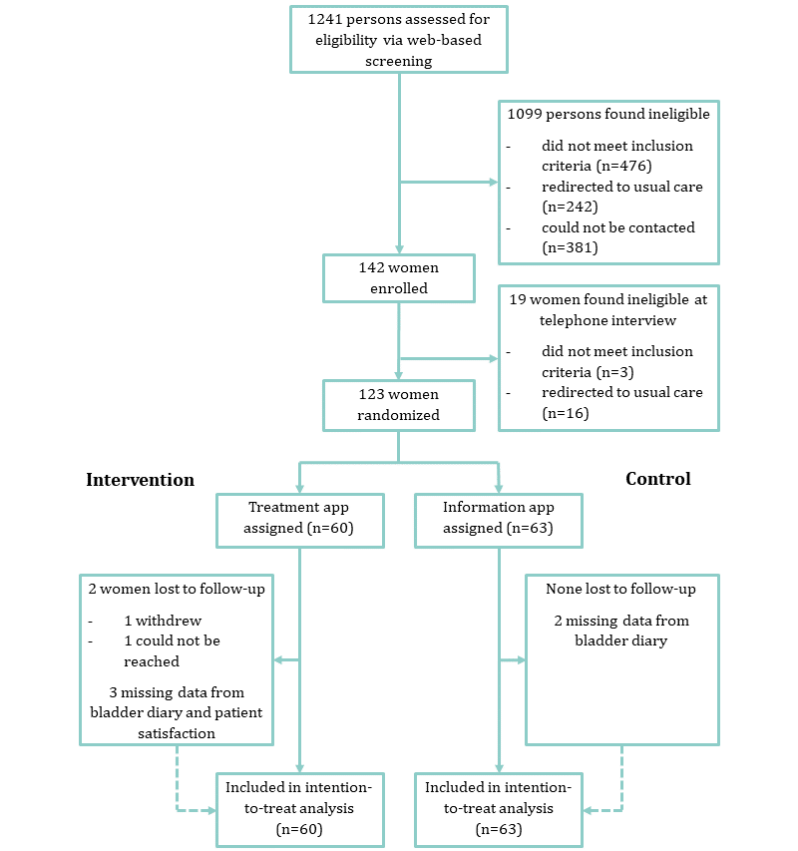
Trial profile.

The web-based screening questionnaire was initiated by 1241 individuals, of whom 1099 were ineligible. A total of 142 women were interviewed, of which 123 were included in the study and randomized to receive either the treatment app (n=60) or the information app (n=63). Two women (both in the treatment group) were lost to follow-up. Five women submitted incomplete follow-up data regarding two secondary outcomes (Figure 2). The median time from randomization to follow-up was 16.1 (IQR 15.0-18.1) weeks in the treatment group and 15.5 (IQR 14.6-17.6) weeks in the information group (*P*=.14).

Baseline characteristics did not differ between the two groups. The mean age of women included was 58 (range 31-77) years. Half of the women (64/123, 52%) were overweight or obese. The majority of participants had received university-level education, and 95% (117/123) of them classified themselves as being quite or very knowledgeable in using computers or tablets. Three-quarters of the participants (88/123, 72%) were diagnosed with MUI, and 69% (85/123) had not previously sought care for their incontinence (Table 2). Severe or very severe incontinence was reported by 38% (47/123) of the participants, and the mean symptom severity score was 11.6 (SD 3.3).

**Table 2:** Baseline characteristics of study participants.

Characteristic				Treatment group (n=60)		Information group (n=63)
**General information**						
	Age in years, mean (SD)			58.9 (9.2)		57.7 (9.9)
	BMI in kg/m^2^, mean (SD)			26.5 (3.6)		25.9 (5.2)
	University education ≥3 years, n (%)			44 (73.3)		35 (55.6)
	Self-rated knowledge in using computers or tablets (1-4 points^a^), mean (SD)			3.5 (0.6)		3.4 (0.6)
	eHEALS^b^ score, mean (SD)			32.9 (5.6)		33.8 (4.5)
**Lifestyle**						
	Physical activity >3 hours/week, n (%)			33 (55.0)		40 (63.5)
	Smoker^c^, n (%)			1 (1.7)		2 (3.2)
	Coffee consumption ≥5 cups/day, n (%)			7 (11.7)		10 (15.9)
	Tea consumption ≥5 cups/day, n (%)			4 (6.7)		2 (3.2)
	Often or always constipated, n (%)			6 (10)		3 (4.8)
**Gynecology**						
	**Parity, n (%)**					
		0	8 (13.3)		7 (11.1)	
		1	4 (6.7)		7 (11.1)	
		≥2	48 (80)		49 (77.8)	
	Postmenopausal >1 year, n (%)			42 (70)		47 (74.6)
	**Current estrogen usage, n (%)**					
		Local estrogen	11 (18.3)		13 (20.6)	
		Systemic estrogen	1 (1.7)		1 (1.6)	
	**Gynecological surgery, n (%)**					
		Hysterectomy	4 (6.7)		7 (11.1)	
		Prolapse surgery	3 (5)		0 (0)	
		Incontinence surgery^d^	3 (5)		3 (4.8)	
**Urinary incontinence**						
	**Symptom diagnosis, n (%)**					
		Mixed urinary incontinence	45 (75)		43 (68.3)	
		Urgency urinary incontinence	15 (25)		20 (31.7)	
	Duration of symptoms >5 years, n (%)			37 (61.7)		38 (60.3)
	Previous health care contact for incontinence symptoms, n (%)			19 (31.7)		19 (30.2)
	ICIQ-UI SF^e^ score, mean (SD)			11.7 (3.5)		11.4 (3.2)
	IEF^f^ per week, mean (SD)^g^			21.8 (16.8)		21.1 (13.7)

^a^A higher score indicates higher self-perceived knowledge.

^b^eHEALS: eHealth Literacy Scale, a self-reported 8-item scale assessing an individual’s ability to identify, evaluate, and use eHealth resources.

^c^No daily smokers, only weekly smokers, participated in the study.

^d^Participants who had undergone incontinence surgery in the last 5 years were not included in the study.

^e^ICIQ-UI SF: International Consultation on Incontinence Questionnaire−Urinary Incontinence Short Form.

^f^IEF: incontinence episode frequency.

^g^Mean (SD) values presented to facilitate comparison with other populations.

### Outcomes

At the 15-week follow-up, women in the treatment group had significantly lower incontinence symptom scores than those in the information group. The estimated between-group difference in mean in the primary outcome, the ICIQ-UI SF score, was −3.1 (95% CI −4.8 to −1.3). Both groups showed an improvement from the baseline but a larger improvement was noted in the treatment group (Table 3).

**Table 3:** Continuous outcomes compared between the treatment group (n=60) and information group (n=63) at follow-up.

Outcome measure and group allocation				Baseline, mean (SD)		Follow-up, mean (SD)		Between-group comparison at follow-up			
							Estimated difference (95% CI)^a^			*P* value	
**Primary outcome**											
	**ICIQ-UI SF^b^**								−3.1 (−4.8 to −1.3)		.001
		Treatment group (n=60)	11.7 (3.5)		7.0 (3.7)^c^						
		Information group (n=63)	11.4 (3.2)		9.8 (3.5)						
**Secondary outcomes**											
	**ICIQ-OAB^d^**								−1.8 (−2.8 to −0.9)		<.001
		Treatment group (n=60)	6.8 (1.8)		4.7 (2.0) ^c^						
		Information group (n=63)	6.7 (1.8)		6.4 (2.0)						
	**ICIQ-LUTSqol^e,f^**								−6.3 (−10.5 to −2.1)		.004
		Treatment group (n=60)	37.6 (8.3)		29.8 (7.8)^c^						
		Information group (n=63)	38.0 (8.1)		36.5 (9.0)						
	**Incontinence Catastrophizing Scale**								−1.6 (−2.8 to −0.3)		.016
		Treatment group (n=60)	4.4 (2.8)		2.3 (2.1)^c^						
		Information group (n=63)	4.7 (2.5)		4.1 (2.5)						

^a^Comparison of mean scores using a linear mixed model.

^b^ICIQ-UI SF: International Consultation on Incontinence Questionnaire−Urinary Incontinence Short Form.

^c^Mean values based on the scores of the 58 treatment app users who completed the follow-up questionnaire.

^d^ICIQ-OAB: International Consultation on Incontinence Questionnaire−Overactive Bladder Module.

^e^ICIQ-LUTSqol: International Consultation on Incontinence Questionnaire−Lower Urinary Tract Symptoms Quality of Life Module.

^f^Three of the items in the ICIQ-LUTSqol included an additional response option, “Not applicable” (these questions concerned partner relations, sex life, and family life). For this study, we set this response option as equal to 1 point, corresponding to the response option “Not at all” (ie, no impact).

Compared with those in the information group, participants in the treatment group also showed significantly greater improvements in the secondary outcomes, with lower scores for urgency symptoms, condition-specific quality of life, and catastrophizing at follow-up. Within-group comparisons revealed statistically significant improvements from baseline to follow-up in all outcomes, except urgency symptoms in the information group (Table 3 and Multimedia Appendix 5).

Participants in both the treatment and information groups exhibited a significant reduction in the number of incontinence episodes (IEF) from the baseline to follow-up. This improvement was greater in the treatment group than in the information group (Table 4). The number of incontinence episodes was reduced by 50% or more in 68% (41/60) of the participants in the treatment group and 30% (19/63) of the participants in the information group. Incontinence aids were used less than once a week at follow-up by the majority of participants (38/60, 63%) in the treatment group compared with those (25/60, 40%) in the information group (Table 5).

**Table 4:** Differences in incontinence episode frequency from the baseline to follow-up compared between the treatment group (n=60) and the information group (n=63).

Group allocation	Baseline, median (IQR)	Follow-up, median (IQR)	Within-group comparison^a^			Between-group comparison at follow-up^b^
			Difference^c^, median (IQR)	*P* value	*P* value	
Treatment app	17.5 (10.5 to 27.1)	3.5 (0.0 to 10.5)	−10.5 (−17.5 to −3.5)	<.001	<.001	
Information app	21.0 (7.0 to 31.5)	10.5 (7.0 to 21.0)	−3.5 (−14.0 to 3.5)	.003		

^a^For within-group comparisons, we calculated the difference from baseline to follow-up for each individual and performed analyses using a Wilcoxon signed-rank test.

^b^Mann-Whitney *U* test.

^c^5 participants in the treatment group and 2 participants in the information group had a missing value at follow-up, and for those, the difference was set to 0 (ie, no change).

**Table 5:** Incontinence aid usage by participants in the treatment group (n=60) and information group (n=63), reported at baseline and at follow-up.

Allocation and incontinence aid usage		Participants, n (%)		*P* value		
		Baseline	Follow-up	Within-group comparison^a^		Between-group comparison at follow-up^b^
**Treatment app**					<.001	.01
	Never	11 (18.3)	26 (43.3)^c^			
	<1/week	10 (16.7)	12 (20)			
	1-3/week	12 (20)	4 (6.7)			
	>3/week, not daily	4 (6.7)	2 (3.3)			
	1/day	12 (20.0)	10 (16.7)			
	>1/day	11 (18.3)	6 (10)^c^			
**Information app**					.15	
	Never	14 (22.2)	14 (22.2)			
	<1/week	11 (17.5)	11 (17.5)			
	1-3/week	7 (11.1)	8 (12.7)			
	>3/week, not daily	4 (6.3)	6 (9.5)			
	1/day	11 (17.5)	13 (20.6)			
	>1/day	16 (25.4)	11 (17.5)			

^a^Wilcoxon signed-rank test.

^b^Mann-Whitney *U* test.

^c^Imputed baseline value for 1 participant lost to follow-up.

**Figure 3:**
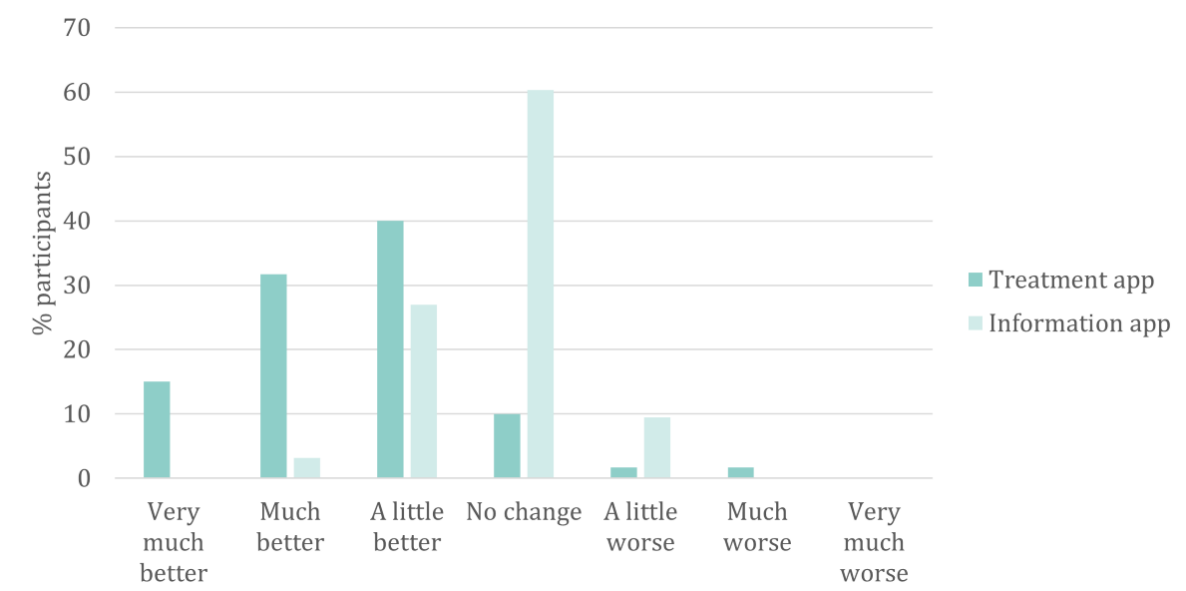
Patient’s Global Impression of Improvement responses reported by the participants at follow-up. Comparison between the treatment group (n=60) and the information group (n=63). *P<.001 (Mann-Whitney U test). Two participants were lost to follow-up in the treatment group and imputed as “no change” for this analysis.

PGI-I scores indicated that 87% (52/60) of the participants in the treatment group reported an improvement as compared with 30% (19/63) in the information group. Figure 3 details the distribution of the responses. Cure was reported by 32% (19/60) of the participants in the treatment group and by 8% (5/63) of the participants in the information group. The OR for cure was 5.4 (95% CI 1.9-15.6, *P*=.002) for the treatment group with the information group as reference. Moreover, the OR for improvement was 15.1 (95% CI 6.0-37.7, *P*<.001) for the treatment group with the information group as reference.

In the treatment group, 7% (4/60) of the participants reported their current satisfaction with the treatment and that they were completely free from urinary leakage and urgency symptoms, whereas 52% (31/60) of the women reported their satisfaction with the treatment despite some remaining symptoms. The remaining 33% (20/60) of the participants reported that they were not satisfied with the treatment, but only 7 of these 20 considered seeking additional care.

### Performance and Adherence

At baseline, 40% (49/123) of the participants were confident that they correctly performed pelvic floor contractions, with no between-group difference. At follow-up, this rate was increased to 62% (37/60) of the participants in the treatment group, and 52% (33/63) of the participants in the information group (*P*=.39). At follow-up, 85% (51/60) of the participants in the treatment group stated that their ability to contract their pelvic floor muscles was slightly or much improved, compared with 16% (10/63) of the participants in the information group (*P*<.001). Similarly, the ability to resist an urge to void was slightly or much improved for 80% (48/60) of the participants in the treatment group, compared with 27% (17/63) of the participants in the information group (*P*<.001).

During the 15-week treatment period, 40 (67%) of the 60 participants in the treatment group used the app more than three times per week, and 6 (10%) participants used it at least three times per day. None of the treatment group participants used other incontinence apps or treatment programs during the study period. In the information group, 2 of the 63 (3%) participants used another PFMT app, and 1 (2%) participant practiced PFMT and tried to resist urgency. Moreover, 1 (2%) participant in the information group sought help from usual care for incontinence-related symptoms during the study period and received incontinence aids, advice on PFMT, and treatment with intravesical hyaluronic acid-chondroitin sulphate. The adherence to the tailored advice is described in Multimedia Appendix 3.

### Technical Issues and User Feedback

No participants reported technical problems with the apps, and no privacy breaches occurred. Many participants in the information group thought that the information was too brief and, therefore, they rarely used the app. Most participants in the treatment group were satisfied with their experience of the app in terms of contents and usability. The users ranked the various treatment app components, with the PFMT portion deemed most useful (ranked 1, 2, or 3 by 95% of all users), followed by the exercise log (ranked 1, 2, or 3 by 80% of all users) and tailored advice (ranked 1, 2, or 3 by 66% of all users).

### Adverse Events

Two participants (both in the treatment group) reported potential adverse events: one of them reported the development of an inguinal hernia during the treatment period, which later required surgery. Discussion of this case with the specialists in the research group, an independent specialist in hernia surgery, and an official at Medical Products Agency, Sweden, led to the conclusion that the hernia was likely not related with the use of the treatment app. The other participant reported altered incontinence symptoms, with decreased urgency but increased episodes of spontaneous urinary leakage.

## Discussion

### Principal Results

In the present randomized controlled trial among women with mixed and urgency urinary incontinence, we found that the treatment app was effective in reducing incontinence symptoms (our primary outcome), with lower scores observed at the 15-week follow-up in the treatment group compared with those in the information group. We also found that treatment app users showed greater improvements regarding quality of life, urgency symptoms, number of incontinence episodes, use of incontinence aids, and catastrophizing, compared with the information app users.

### Clinical Relevance

About 87% (52/60) of the treatment app users reported an improvement, and half of them (28/60, 47%) reported *much* or *very much* improvement. A 50% reduction in the frequency of incontinence episodes is considered clinically relevant, and this degree of reduction was observed for the majority of the treatment app users in our study [32]. For women with SUI, reductions of 2.5 points in the ICIQ-UI SF score and 3.7 points in the ICIQ-LUTSqol score has been considered to reflect clinically relevant improvement after pelvic floor muscle training via an eHealth approach [22]. The within-group reductions observed in our study were larger than these thresholds and also larger than the 4- and 6-point reduction in the ICIQ-UI SF and ICIQ-LUTSqol scores established by Lim et al as minimum important differences for other conservative management [33]. To our knowledge, there are no similar studies of women with UUI and MUI, but minimum important differences are likely to be at a similar level compared with the studies mentioned above. The PGI-I and comparison with minimum important differences indicate that the changes in symptoms and quality of life for the treatment group observed in our study are clinically relevant.

### Strengths and Limitations

One of the strengths of our study was that we used clinically relevant outcomes that were carefully selected to cover different aspects of UUI and MUI, including symptoms, quantification, quality of life, and subjective improvement. The three ICIQ scores, including the one used as the primary outcome (ICIQ-UI SF), are all Grade A or A+ recommended outcome measures according to the International Continence Society [3]. These questionnaires are also validated for electronic use [34,35]. Additionally, our study was adequately sized and conducted with external monitoring, as a mark of quality. There were very small losses to follow-up and no internal losses, except for the secondary outcome of IEF. We developed and utilized an algorithm to identify suitable participants, and all participants experienced extensive incontinence symptoms at the baseline, strengthening the need for treatment. Another strength of our study was that the app was thoroughly designed and stable and required no updates during the study period. No users experienced technical issues. The treatment app included a patient-centered design, such that the user herself decided which parts of the app to use. Tailored advice, based on information about the user’s lifestyle and incontinence symptoms, offered guidance on what might be the most beneficial component or feature of the app for her to focus on. All participants in the treatment group downloaded and activated the app, and most of them regularly used the app. The risk for contamination between the two groups was negligible since activation of the treatment app required a unique one-time authorization code. The treatment app featured information and exercises covering multiple topics related to UUI and MUI, resembling a clinical reality with a multi-faceted intervention. Therefore, this study cannot discriminate how different parts of the app contributed to the various effects.

A potential limitation of this study is that we cannot yet assess the long-term effects of the app. However, we previously reported that our smartphone app targeted at women with SUI had a long-lasting effect at the 2-year follow-up [21]. Similar to most other investigations of eHealth or behavioral therapy interventions, another limitation of our study was that the participants could not be blinded to their group allocation. Our choice to not use care-as-usual as a control group could also be viewed as a limitation. However, seeking care-as-usual was not a likely option for most of our intended target population; thus, we argue that the information app was the most comparable control. Another potential concern is the lack of face-to-face contact with a health care provider and that there was no professional assessment of the participants’ ability to perform correct PFMT contractions. However, in a JAMA review from 2017, unsupervised PFMT is recommended as a first-line treatment after exclusion of serious underlying pathologies [9]. The treatment app included information on how to correctly perform contractions, and it recommended women who were uncertain of their contraction technique to contact their ordinary health care provider for advice. At follow-up, no treatment app users had sought help from usual care, and the majority stated that their ability to perform PFMT contractions had improved. Most of the women in the treatment group were satisfied with the treatment, and of those who were not satisfied, only a few intended to seek care elsewhere. However, since the qualitative feedback was optional, we do not know the reason behind their decision not to seek further care despite not being satisfied. Furthermore, this study focused on the presentation of quantitative data, and while this is a strength when investigating efficacy, a deeper analysis of the qualitative data collected might provide valuable information about the experiences of the participants in the trial. A common issue in research, and particularly in studies of eHealth interventions, is that the participants’ education level is often higher than that of the general population. The average education level of people in Sweden is higher than that of people in many other countries; nonetheless, women with a university-level education were over-represented among our participants, which may potentially affect the generalizability of our results.

### Comparison With Prior Work

Several systematic reviews report that antimuscarinic drugs and mirabegron yield a mean reduction corresponding to half a leakage per 24 hours at the 3-month follow-up when compared with the placebo [36-39]. The median reduction for the treatment group in our study was twice as large as this. Moreover, antimuscarinic medication is commonly associated with side-effects, such as dry mouth and constipation. Mirabegron is better tolerated but has side-effects, such as urinary tract infections, irregular heartrate, and palpitations [3,36,37]. In contrast, behavioral treatment and lifestyle advice carry no known side-effects. Women with urgency urinary incontinence are more likely to achieve improvement, cure, and satisfaction with behavioral therapy than with anticholinergics [40]. The cure and improvement rates in our treatment app group were in line with the findings from other studies. A systematic review update from 2018 reported a 25%-30% cure rate of urgency urinary incontinence with neuromodulation, behavioral therapy, or combined anticholinergic and behavioral therapy. The OR for cure of urgency incontinence with behavioral therapy was 2.75 (95% CI 1.53-4.92) compared with the placebo, sham, or no treatment [40].

### Conclusions and Outlook

UUI and MUI affect many women and can have a potentially large impact on their quality of life. Thus, it is important to offer effective treatment options that can reach many patients, and eHealth methods are a new potential means of supporting self-management. Providing treatment that does not require face-to-face contact with the health care service provider might facilitate increased care-seeking among these women. Additionally, eHealth tools (eg, smartphone apps) provide possibilities for adherence-promotion, such as reminder notifications, and can be tailored for the specific user, such as through the tailored advice provided in our presently tested treatment app. Concerns have recently been raised about a digital divide such that some groups might be less able to use these digital health aids [41-43]. Future research is needed to identify ways to improve the interventions, or the development process, to make eHealth treatment options more accessible or relevant for new user groups. Further investigations are also needed to evaluate the use of algorithms to select patients for self-management of these conditions, with regard to medical safety. For populations similar to the participants in our present study, our results indicate that the treatment app is already an effective treatment option. There remains a need to study the long-term effects, and to decide how to make this app available to patients—that is, whether the app can be offered as a stand-alone, first-line intervention for women with an uncomplicated medical history, or whether it should be regulated and prescribed only by health care professionals.

To our knowledge, this is the first study to demonstrate the potential to provide an effective, tailored, app-based treatment to women with urgency or mixed urinary incontinence suited for self-management. Our results show an efficacy that is comparable to other first-line treatments available. Therefore, we propose that this app could be added to the treatment options offered as part of usual care for women presenting with these conditions.

## Appendix

Multimedia Appendix 1 Algorithm with structured questions regarding red-flag symptoms.

Multimedia Appendix 2 Information on the treatment program delivered via the Tät II treatment app.

Multimedia Appendix 3 Criteria, tailored advice, and participant adherence data for treatment information delivered via the Tät II treatment app.

Multimedia Appendix 4 Data collection timeline.

Multimedia Appendix 5 Continuous outcomes compared between the treatment group (n=60) and the information group (n=63) at follow-up, and within-group comparisons from baseline to follow-up.

Multimedia Appendix 6 CONSORT-EHEALTH checklist (V 1.6.1).

## Abbreviations

eHEALS: eHealth Literacy Scale
IC Scale: Incontinence Catastrophizing Scale
ICIQ-LUTSqol: International Consultation on Incontinence Questionnaire−Lower Urinary Tract Symptoms Quality of Life Module
ICIQ-OAB: International Consultation on Incontinence Questionnaire−Overactive Bladder Module
ICIQ-UI SF: International Consultation on Incontinence Questionnaire−Urinary Incontinence Short Form
IEF: incontinence episode frequency
MUI: mixed urinary incontinence
OR: odds ratio
PFMT: pelvic floor muscle training
PGI-I: Patient Global Impression of Improvement
SUI: stress urinary incontinence
UUI: urgency urinary incontinence

## References

[1] Hannestad YS, Rortveit G, Sandvik H, Hunskaar S (2000). A community-based epidemiological survey of female urinary incontinence: the Norwegian EPINCONT study. Epidemiology of Incontinence in the County of Nord-Trøndelag. J Clin Epidemiol.

[2] Hunskaar S, Burgio K, Diokno A, Herzog AR, Hjälmås K, Lapitan MC (2003). Epidemiology and natural history of urinary incontinence in women. Urology.

[3] Abrams P, Cardozo L, Wagg A, Wein A (2017). Incontinence 6th Edition.

[4] Milsom I, Coyne KS, Nicholson S, Kvasz M, Chen C, Wein AJ (2014). Global prevalence and economic burden of urgency urinary incontinence: a systematic review. Eur Urol.

[5] Haylen BT, de Ridder D, Freeman RM, Swift SE, Berghmans B, Lee J, Monga A, Petri E, Rizk DE, Sand PK, Schaer GN, International UA, International CS (2010). An International Urogynecological Association (IUGA)/International Continence Society (ICS) joint report on the terminology for female pelvic floor dysfunction. Neurourol Urodyn.

[6] Siddiqui NY, Levin PJ, Phadtare A, Pietrobon R, Ammarell N (2014). Perceptions about female urinary incontinence: a systematic review. Int Urogynecol J.

[7] Coyne KS, Sexton CC, Irwin DE, Kopp ZS, Kelleher CJ, Milsom I (2008). The impact of overactive bladder, incontinence and other lower urinary tract symptoms on quality of life, work productivity, sexuality and emotional well-being in men and women: results from the EPIC study. BJU Int.

[8] Coyne KS, Zhou Z, Thompson C, Versi E (2003). The impact on health-related quality of life of stress, urge and mixed urinary incontinence. BJU Int.

[9] Lukacz ES, Santiago-Lastra Y, Albo ME, Brubaker L (2017). Urinary Incontinence in Women: A Review. JAMA.

[10] National Institute for Health and Care Excellence (NICE) (2019). NICE Guidance - Urinary incontinence and pelvic organ prolapse in women: management. BJU Int.

[11] Bo K, Fernandes A, Duarte T, Brito L, Ferreira C (2020). Is pelvic floor muscle training effective for symptoms of overactive bladder in women? A systematic review. Physiotherapy.

[12] Minassian VA, Yan X, Lichtenfeld MJ, Sun H, Stewart WF (2012). The iceberg of health care utilization in women with urinary incontinence. Int Urogynecol J.

[13] Hägglund D, Wadensten B (2007). Fear of humiliation inhibits women's care-seeking behaviour for long-term urinary incontinence. Scand J Caring Sci.

[14] Bernard S, Boucher S, McLean L, Moffet H (2019). Mobile technologies for the conservative self-management of urinary incontinence: a systematic scoping review. Int Urogynecol J.

[15] Brown J, Michie S, Geraghty AWA, Yardley L, Gardner B, Shahab L, Stapleton JA, West R (2014). Internet-based intervention for smoking cessation (StopAdvisor) in people with low and high socioeconomic status: a randomised controlled trial. Lancet Respir Med.

[16] Massoudi B, Holvast F, Bockting CLH, Burger H, Blanker MH (2019). The effectiveness and cost-effectiveness of e-health interventions for depression and anxiety in primary care: A systematic review and meta-analysis. J Affect Disord.

[17] Novara G, Checcucci E, Crestani A, Abrate A, Esperto F, Pavan N, De Nunzio C, Galfano A, Giannarini G, Gregori A, Liguori G, Bartoletti R, Porpiglia F, Scarpa RM, Simonato A, Trombetta C, Tubaro A, Ficarra V (2020). Telehealth in Urology: A Systematic Review of the Literature. How Much Can Telemedicine Be Useful During and After the COVID-19 Pandemic?. European Urology.

[18] Grimes CL, Balk EM, Crisp CC, Antosh DD, Murphy M, Halder GE, Jeppson PC, Weber LeBrun EE, Raman S, Kim-Fine S, Iglesia C, Dieter AA, Yurteri-Kaplan L, Adam G, Meriwether KV (2020). A guide for urogynecologic patient care utilizing telemedicine during the COVID-19 pandemic: review of existing evidence. Int Urogynecol J.

[19] Sudol NT, Adams-Piper E, Perry R, Lane F, Chen KT (2019). In Search of Mobile Applications for Patients With Pelvic Floor Disorders. Female Pelvic Med Reconstr Surg.

[20] Asklund I, Nyström E, Sjöström M, Umefjord G, Stenlund H, Samuelsson E (2016). Mobile app for treatment of stress urinary incontinence: a randomized controlled trial. Neurourol Urodyn.

[21] Hoffman V, Söderström L, Samuelsson E (2017). Self-management of stress urinary incontinence via a mobile app: two-year follow-up of a randomized controlled trial. Acta Obstet Gynecol Scand.

[22] Nyström E, Sjöström M, Stenlund H, Samuelsson E (2015). ICIQ symptom and quality of life instruments measure clinically relevant improvements in women with stress urinary incontinence. Neurourol Urodyn.

[23] Sjöström M, Lindholm L, Samuelsson E (2017). Mobile App for Treatment of Stress Urinary Incontinence: A Cost-Effectiveness Analysis. J Med Internet Res.

[24] Sjöström M, Umefjord G, Stenlund H, Carlbring P, Andersson G, Samuelsson E (2015). Internet-based treatment of stress urinary incontinence: 1- and 2-year results of a randomized controlled trial with a focus on pelvic floor muscle training. BJU Int.

[25] Huang AJ, Hess R, Arya LA, Richter HE, Subak LL, Bradley CS, Rogers RG, Myers DL, Johnson KC, Gregory WT, Kraus SR, Schembri M, Brown JS (2012). Pharmacologic treatment for urgency-predominant urinary incontinence in women diagnosed using a simplified algorithm: a randomized trial. Am J Obstet Gynecol.

[26] Avery K, Donovan J, Peters TJ, Shaw C, Gotoh M, Abrams P (2004). ICIQ: a brief and robust measure for evaluating the symptoms and impact of urinary incontinence. Neurourol Urodyn.

[27] Klovning A, Avery K, Sandvik H, Hunskaar S (2009). Comparison of two questionnaires for assessing the severity of urinary incontinence: The ICIQ-UI SF versus the incontinence severity index. Neurourol Urodyn.

[28] Abrams P, Avery K, Gardener N, Donovan J, ICIQ Advisory Board (2006). The International Consultation on Incontinence Modular Questionnaire: www.iciq.net. J Urol.

[29] Sullivan MJL, Bishop SR, Pivik J (1995). The Pain Catastrophizing Scale: Development and validation. Psychological Assessment.

[30] Yalcin I, Bump RC (2003). Validation of two global impression questionnaires for incontinence. Am J Obstet Gynecol.

[31] Albers-Heitner PCP, Lagro-Janssen TALM, Joore MMA, Berghmans BLCM, Nieman FF, Venema PPL, Severens JJL, Winkens RRAG (2011). Effectiveness of involving a nurse specialist for patients with urinary incontinence in primary care: results of a pragmatic multicentre randomised controlled trial. Int J Clin Pract.

[32] Shamliyan T, Wyman JF, Ramakrishnan R, Sainfort F, Kane RL (2012). Benefits and harms of pharmacologic treatment for urinary incontinence in women: a systematic review. Ann Intern Med.

[33] Lim R, Liong ML, Lim KK, Leong WS, Yuen KH (2019). The Minimum Clinically Important Difference of the International Consultation on Incontinence Questionnaires (ICIQ-UI SF and ICIQ-LUTSqol). Urology.

[34] Sjöström M, Stenlund H, Johansson S, Umefjord G, Samuelsson E (2012). Stress urinary incontinence and quality of life: a reliability study of a condition-specific instrument in paper and web-based versions. Neurourol Urodyn.

[35] Uren AD, Cotterill N, Parke SE, Abrams P (2017). Psychometric equivalence of electronic and telephone completion of the ICIQ modules. Neurourol Urodyn.

[36] Nabi G, Cody JD, Ellis G, Herbison P, Hay-Smith J (2006). Anticholinergic drugs versus placebo for overactive bladder syndrome in adults. Cochrane Database Syst Rev.

[37] (2013). Mirabegron for treating symptoms of overactive bladder - Technology appraisal guidance TA290. National Institute for HealthCare Excellence (NICE).

[38] Samuelsson E, Odeberg J, Stenzelius K, Molander U, Hammarström M, Franzen K, Andersson G, Midlöv P (2015). Effect of pharmacological treatment for urinary incontinence in the elderly and frail elderly: A systematic review. Geriatr Gerontol Int.

[39] Sexton CC, Notte SM, Maroulis C, Dmochowski RR, Cardozo L, Subramanian D, Coyne KS (2011). Persistence and adherence in the treatment of overactive bladder syndrome with anticholinergic therapy: a systematic review of the literature. Int J Clin Pract.

[40] Balk E, Adam G, Kimmel H, Rofeberg V, Saeed I, Jeppson P, Trikalinos T (2018). Nonsurgical treatments for urinary incontinence in women: a systematic review update [Internet]. AHRQ Comparative Effectiveness Reviews. Report No. 212 (Prepared by the Brown Evidence-based Practice Center under Contract No. 290-2015-00002-I for AHRQ and PCORI).

[41] Kontos E, Blake KD, Chou WS, Prestin A (2014). Predictors of eHealth usage: insights on the digital divide from the Health Information National Trends Survey 2012. J Med Internet Res.

[42] Latulippe K, Hamel C, Giroux D (2017). Social Health Inequalities and eHealth: A Literature Review With Qualitative Synthesis of Theoretical and Empirical Studies. J Med Internet Res.

[43] Neter E, Brainin E (2012). eHealth literacy: extending the digital divide to the realm of health information. J Med Internet Res.

