# Supplementary figures and images for: A Mobile App for Self-management of Urgency and Mixed Urinary Incontinence in Women: Randomized Controlled Trial
Source: J Med Internet Res. 2021 Apr 5;23(4):e19439. doi: 10.2196/19439 (PMC8056293; doi:10.2196/19439)

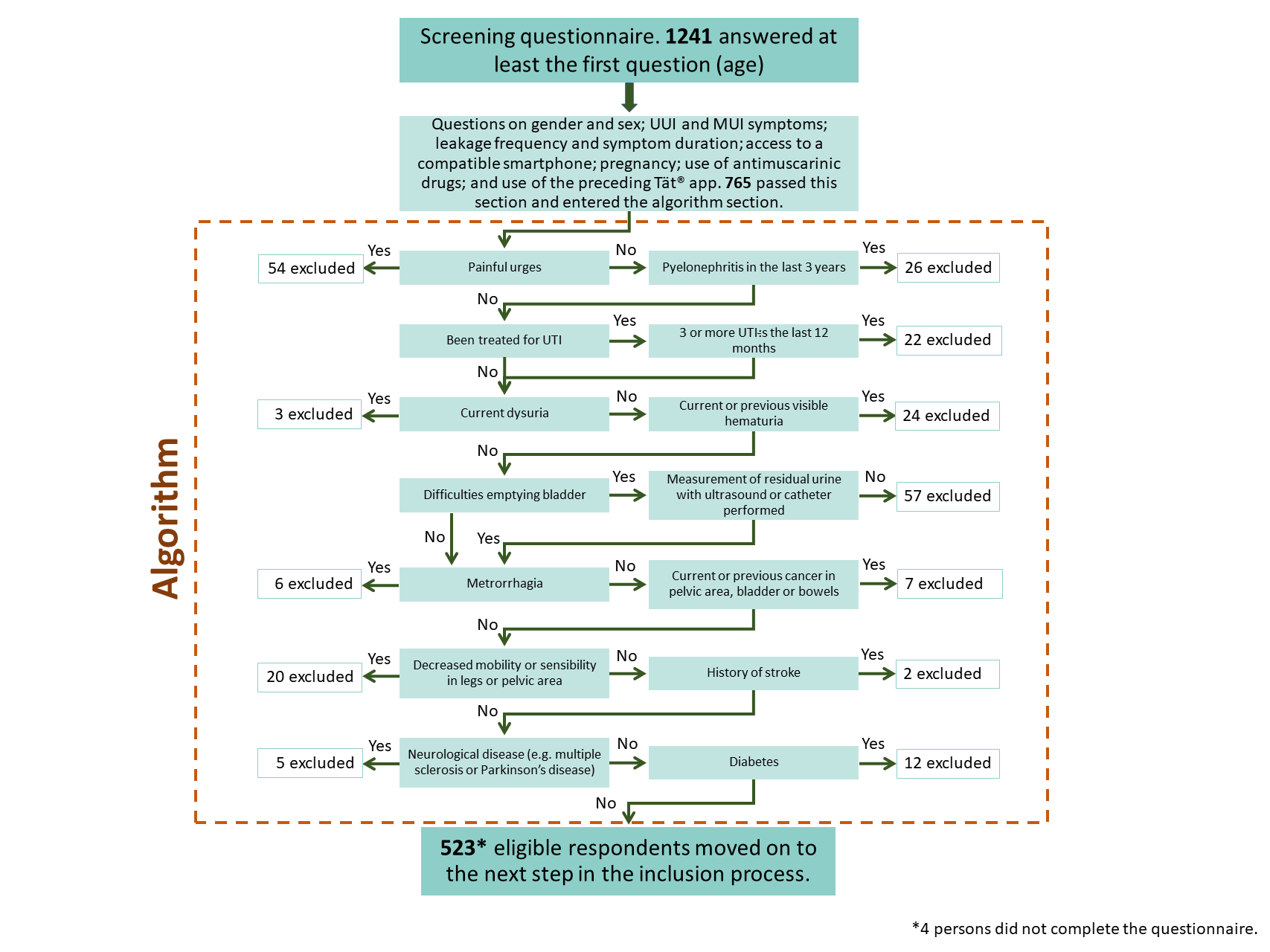

Supplement: Multimedia Appendix 1 [file jmir_v23i4e19439_app1.png]

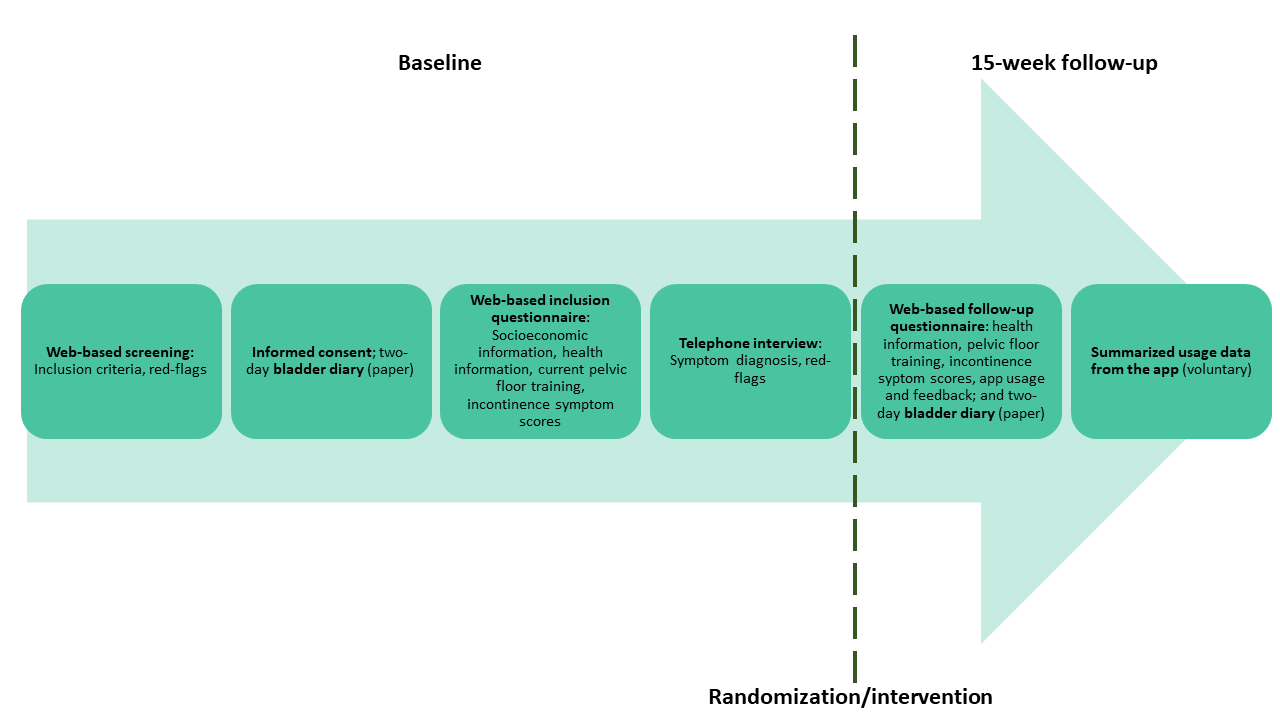

Supplement: Multimedia Appendix 4 [file jmir_v23i4e19439_app4.png]
