# Supplementary material for: A Mobile App for Self-management of Urgency and Mixed Urinary Incontinence in Women: Randomized Controlled Trial
Source: J Med Internet Res. 2021 Apr 5;23(4):e19439. doi: 10.2196/19439 (PMC8056293; doi:10.2196/19439)
Supplement: Multimedia Appendix 2 [file jmir_v23i4e19439_app2.doc]

# Information on the treatment programs in the Tät II treatment app

## Pelvic floor muscle training (PFMT)

- Information (text and visuals) about the anatomy of the pelvic floor
- Information (text and visuals) about PFMT and how to find the correct muscles
- Preparatory exercises to practice different types of contractions: find the correct muscles, strength contractions, endurance contractions, quick contractions (“the knack”).
- An exercise program with increasing difficulty at six basic and five advanced levels.
- Graphic bar visualising the duration and intensity of each contraction.

### Example of an exercise:

**Basic level 6**

10 strength contractions, 1 endurance contraction (35 seconds), 5 quick contractions

Exercise while standing up, three times a day.

Once you have learned the contractions, continue exercising until the end of the three-month period or go on to the advanced exercises after 1-2 weeks.


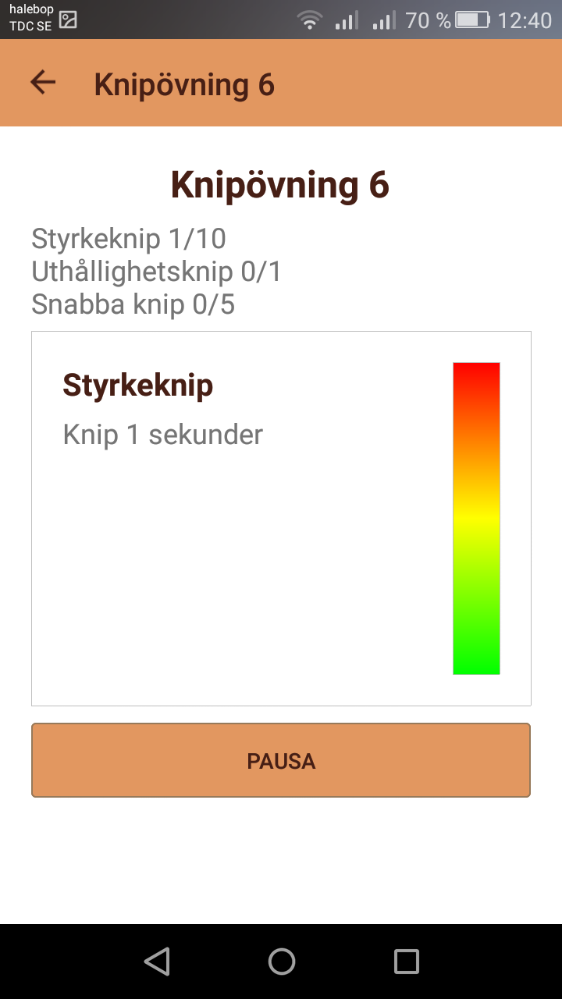
Use the contractions in everyday life as well: a quick contraction before coughing, a strength contraction while lifting, and an endurance contraction while walking.

## Bladder training

- Information (text and visuals) about bladder physiology
- Information (text and visuals) about bladder training, preventive voiding, a toilet relaxation exercise, and tips on how to endure urgency
- An exercise program with increasing difficulty at 7 levels^a^
- A timer indicating the length of the exercise (i.e., for how long to endure the urgency) and a graphic bar to aid endurance contractions

^a^The exercise program is focused on enduring urgency to postpone voiding. It does not feature scheduled voiding.

### Examples of two exercises:

**Bladder exercise 1**

When an urge to void occurs, make an endurance contraction for as long as you can. Try squeezing your toes for strength. If the urge recurs, repeat the contraction. Delay voiding for 60 seconds.

Use the tips in your training.


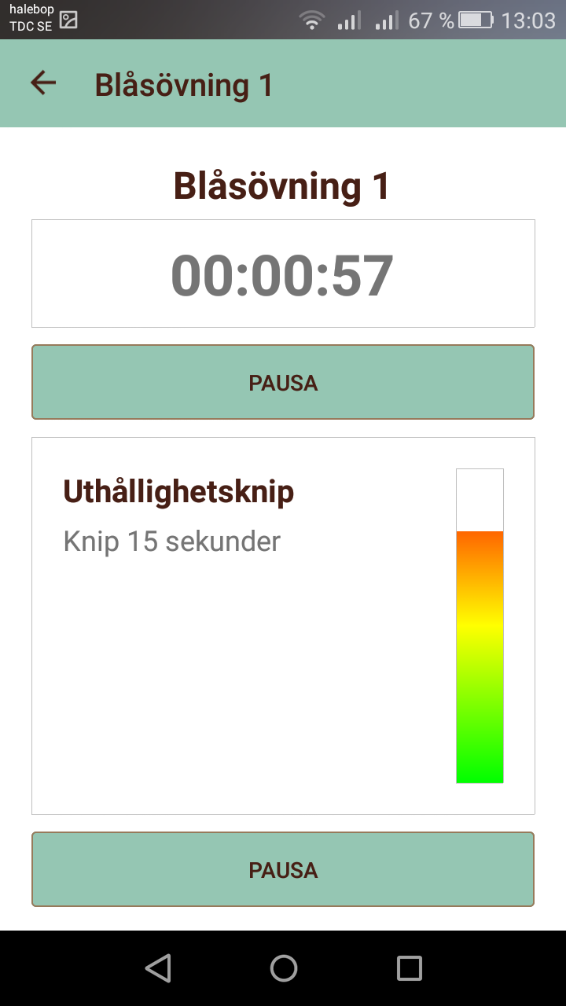


**Bladder exercise 6: Running tap water**

Turn on a tap and let the water run. When an urge to void occurs, make an endurance contraction for as long as you can. Try squeezing your toes for strength. If the urge recurs, repeat the contraction. Delay voiding for 15 minutes.

Use the tips in your training.

## Psycho-education

- Information (text, visuals, and examples) on the psychology of urgency
- Three consecutive self-managed tasks focusing on thought, body and behavior; prevention and avoidance; and finding alternative strategies
- No therapeutical feedback given

### Example of a task:

#### Task 1: Thought, body, behavior

In this task, try to think of one or more situations where you worry about, or fear, leakage. The focus is your worry of leakage, not an actual leakage. Choose 2-3 such situations in the next week and describe your experiences. If you have difficulty finding a specific situation, try the “running tap” situation. Turn on a tap and let the water run and try to pay attention to your feelings.

Observe and describe

- The situation
- The degree of worry (between 0-10)
- Your thoughts (For example: When you experience urgency, what are your thoughts? What did you tell yourself when hurrying to the toilet? When with other people, what do you tell yourself about your personal hygiene or how you look?)
- The sensation in your body
- Your strategy for dealing with the situation


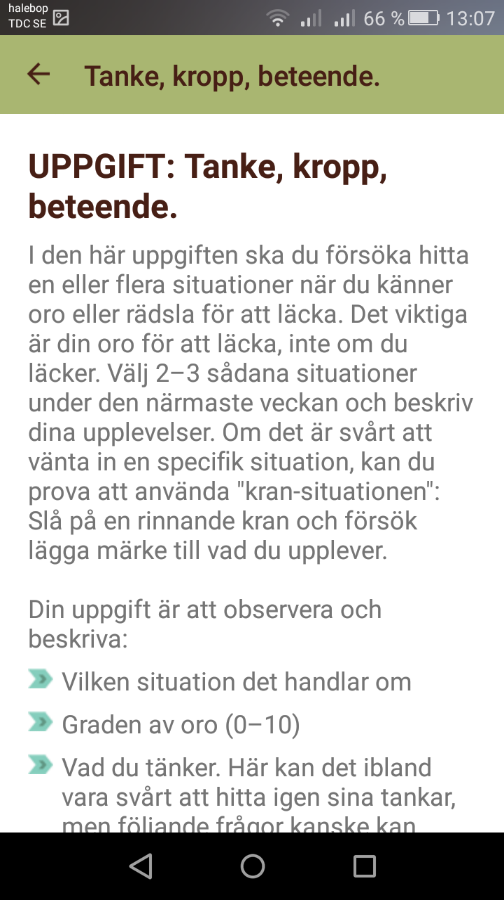

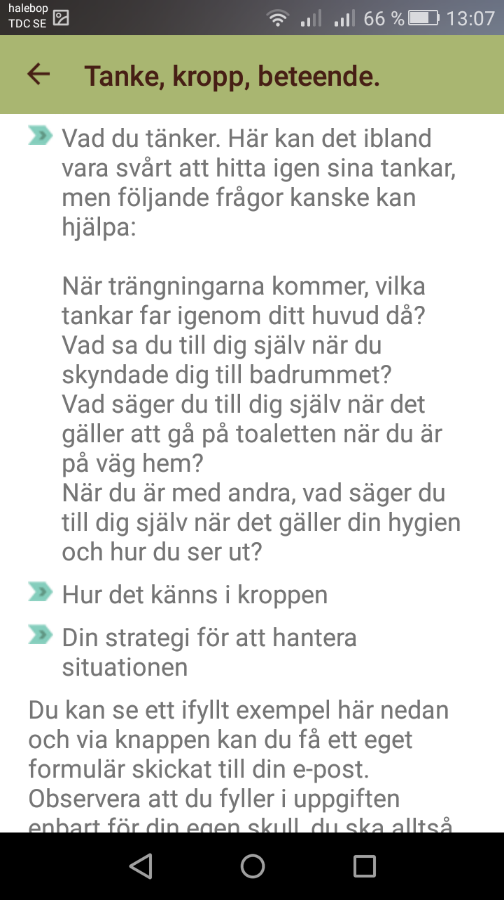

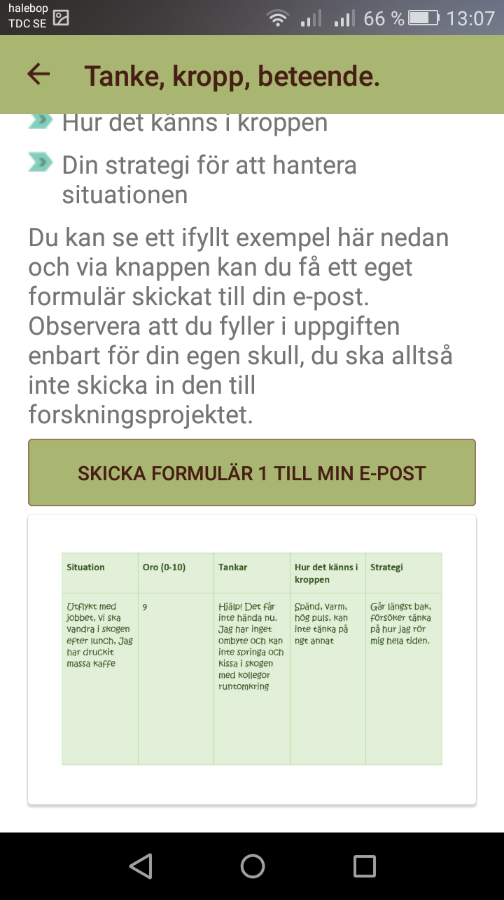


## Automated reinforcement messages

- Self-assessment of bother from leakage, urges and fear of leakage at start, at 4 weeks, 8 weeks and at 12 weeks (0=no bother, 10=very much bother)
- The reinforcement messages are automated in-app responses based on the change in level of bother compared to the previous self-assessment
- For each of the categories (leakage, urges, and fear of leakage) the responses include the change in level of bother and a specific advice regarding how to continue with the different treatment programs in the app
- The most recent reinforcement message in each category is accessible in the app until the next self-assessment

### Example of a reinforcement message:

#### Urgency

Unchanged

Your degree of bother from urgency has not changed. Have you read the section “My advice”? Continue to do your exercises. It usually takes 1-2 months until you experience the full results of the training and lifestyle changes.

**
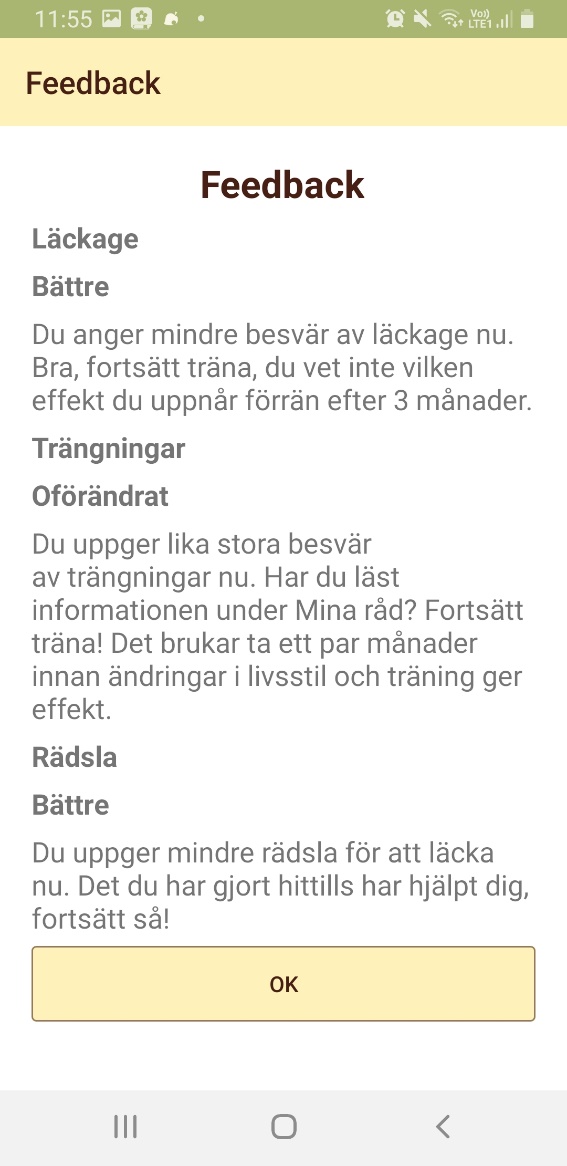

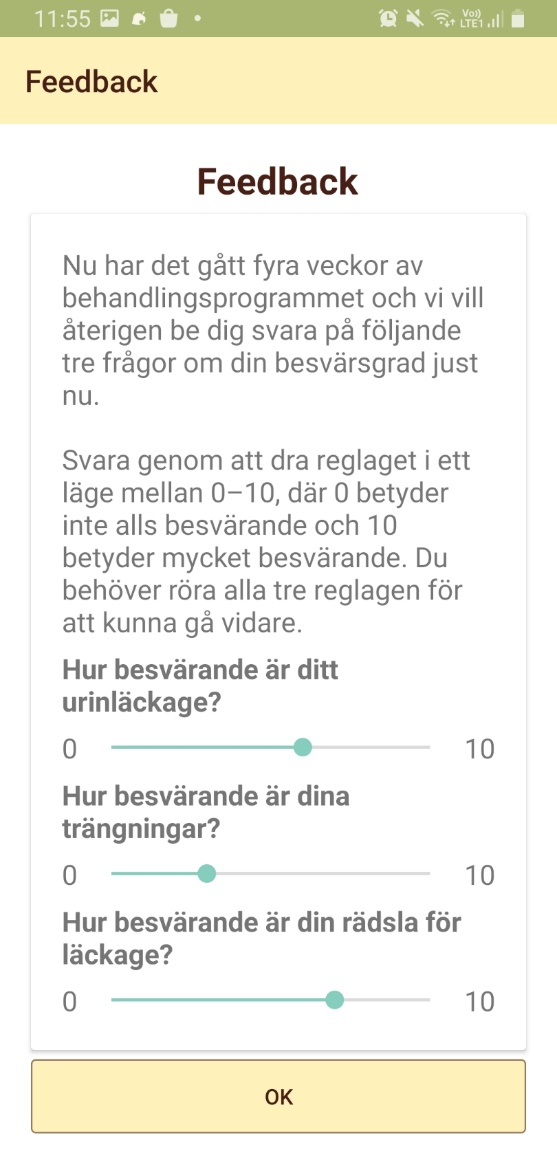
**
