# Supplementary material for: A Mobile App for Self-management of Urgency and Mixed Urinary Incontinence in Women: Randomized Controlled Trial
Source: J Med Internet Res. 2021 Apr 5;23(4):e19439. doi: 10.2196/19439 (PMC8056293; doi:10.2196/19439)
Supplement: Multimedia Appendix 3 [file jmir_v23i4e19439_app3.doc]

# Criteria, tailored advice, and participant adherence data for treatment information delivered via the Tät II treatment app.

| **Criteria** | | **Advice** | | **Criteria for adherence^a^** | **Received the advice,**  **n** | | **Followed the advice,  n (%)** | **Lost to follow-up,**  **n** |
| --- | --- | --- | --- | --- | --- | --- | --- | --- |
| Urgency urinary incontinence | | **1a** | Urgency urinary incontinence diagnosis. PFMT^b^ recommended. | Regular PFMT^b^ training 1-3 times per week or more | 15 | | 9 (60) | 2 |
| Mixed urinary incontinence | | **1b** | Mixed urinary incontinence diagnosis. PFMT^b^ recommended. |  | 45 | | 37 (82) | 0 |
| Micturition volume (total, day+night) | >2 litres | **2a** | Reduced intake of fluid | Reduced intake of fluid | 20 | | 4 (20) | 0 |
|  | 1 litre or less | **2b** | Increased intake of fluid | Increased intake of fluid | 2 | | 1 (50) | 1 |
| Micturition volume (average portion) | 2 decilitres or less | **3** | Bladder training | Regular bladder training 1-3 times per week or more | 21 | | 14 (67) | 2 |
| Average >3 occasions of "preventive" micturition per day | | **4** | Reduced "preventive" micturition | Reduced "preventive" micturition | 5 | | 5 (100) | 0 |
| Age >40 and menopause or age >55 and unknown menstrual state | | **5** | Try local estrogen treatment | Begun, continued or increased use of local estrogen | 42 | | 20 (48) | 2 |
| A total of 5 or more cups per day of either coffee or tea | | **6** | Reduced intake of coffee and tea | Reduced intake of coffee or tea | 36 | | 8 (22) | 1 |
| BMI^c^ >25 | | **7** | Weight reduction | 5% or more weight reduction | 34 | | 1 (3) | 2 |
| Smoker | | **8** | Smoking cessation | No smoking | 1 | | - | 1 |
| >6 points on the IC Scale^d^ | | **9** | Use the psychology part | Completion of 1 or more psychology tasks | 14 | | 4 (29) | 0 |
| Symptoms of constipation | | **10** | Regular physical activity, food rich in fibres, normal fluid intake | No, or reduced, constipation symptoms | 18 | | 8 (44) | 1 |
| ^a^Based on information from the follow-up questionnaire. ^b^PFMT=pelvic floor muscle training. ^c^BMI=body mass index. ^d^IC Scale=Incontinence Catastrophizing Scale. | | | | | |  | | |
