# Supplementary material for: A Mobile App for Self-management of Urgency and Mixed Urinary Incontinence in Women: Randomized Controlled Trial
Source: J Med Internet Res. 2021 Apr 5;23(4):e19439. doi: 10.2196/19439 (PMC8056293; doi:10.2196/19439)
Supplement: Multimedia Appendix 5 [file jmir_v23i4e19439_app5.doc]

# Continuous outcomes compared between the treatment group (n=60) and the information group (n=63) at follow-up, and within-group comparisons from baseline to follow-up.

| **Outcome measure** | | **Group allocation** | **Baseline Mean (SD)** | **Follow-up Mean (SD)** | **Within-group comparison** | | **Between-group comparison at follow-up** | |
| --- | --- | --- | --- | --- | --- | --- | --- | --- |
|  |  |  |  |  | **Mean difference  (95% confidence interval)^a^** | ***P* value** | **Estimated difference (95% confidence interval)^b^** | ***P* value** |
| *Primary outcome* | **ICIQ-UI SF^c^** | *Treatment group***^d^** | 11.7 (3.5) | 7.0 (3.7)^e^ | -4.7 (-5.7 to -3.7) | <.001 | -3.1 (-4.8 to -1.3) | .001 |
|  |  | *Information group***^f^** | 11.4 (3.2) | 9.8 (3.5) | -1.6 (-2.3 to -1.0) | <.001 |  |  |
| *Secondary outcomes* | **ICIQ-OAB^g^** | *Treatment group***^d^** | 6.8 (1.8) | 4.7 (2.0)^e^ | -2.1 (-2.5 to -1.6) | <.001 | -1.8 (-2.8 to -.9) | <.001 |
|  |  | *Information group***^f^** | 6.7 (1.8) | 6.4 (2.0) | -.2 (-.6 to .2) | .243 |  |  |
|  | **ICIQ-LUTSqol**^h,i^ | *Treatment group***^d^** | 37.6 (8.3) | 29.8 (7.8)^e^ | -7.7 (-9.2 to -6.2) | <.001 | -6.3 (-10.5 to -2.1) | .004 |
|  |  | *Information group***^f^** | 38.0 (8.1) | 36.5 (9.0) | -1.5 (-2.9 to -.2) | .031 |  |  |
|  | **Incontinence Catastrophizing Scale** | *Treatment group***^d^** | 4.4 (2.8) | 2.3 (2.1)^e^ | -2.2 (-2.9 to -1.5) | <.001 | -1.6 (-2.8 to -.3) | .016 |
|  |  | *Information group***^f^** | 4.7 (2.5) | 4.1 (2.5) | -.6 (-1.2 to -.0) | .037 |  |  |
| ^a^Comparison using a Student’s t-test ^b^Comparison using a linear mixed model. ^c^ICIQ-UI SF=International Consultation on Incontinence Questionnaire (ICIQ) Urinary Incontinence Short Form. ^d^N=60. ^e^Mean values based on the scores of the 58 treatment app users who completed the follow-up questionnaire. ^f^N=63. ^g^ICIQ-OAB=ICIQ Overactive Bladder Module. ^h^ICIQ-LUTSqol=ICIQ Lower Urinary Tract Symptoms Quality of Life Module. ^i^Three of the items in the ICIQ-LUTSqol included an additional response option, “not applicable” (these questions concerned partner relations, sex life, and family life). For this study, we set this response option as equal to 1 point, corresponding to the response option “not at all” (ie, no impact). | | | | | | | | |
